# Drug Delivery Systems in the Development of Novel Strategies for Glioblastoma Treatment

**DOI:** 10.3390/pharmaceutics14061189

**Published:** 2022-06-01

**Authors:** Wiam El Kheir, Bernard Marcos, Nick Virgilio, Benoit Paquette, Nathalie Faucheux, Marc-Antoine Lauzon

**Affiliations:** 1 Advanced Dynamic Cell Culture Systems Laboratory, Department of Chemical Engineering and Biotechnology Engineering, Faculty of Engineering, Université de Sherbrooke, 2500 Boul. Université, Sherbrooke, QC J1K 2R1, Canada; wiam.el.kheir@usherbrooke.ca; 2 Laboratory of Cell-Biomaterial Biohybrid Systems, Department of Chemical Engineering and Biotechnology Engineering, Faculty of Engineering, Université de Sherbrooke, 2500 Boul. Université, Sherbrooke, QC J1K 2R1, Canada; nathalie.faucheux@usherbrooke.ca; 3 Department of Chemical Engineering and Biotechnology Engineering, Faculty of Engineering, Université de Sherbrooke, 2500 Boul. Université, Sherbrooke, QC J1K 2R1, Canada; bernard.marcos@usherbrooke.ca; 4 Department of Chemical Engineering, Polytechnique Montréal, 2500 Chemin de Polytechnique, Montréal, QC H3T 1J4, Canada; nick.virgilio@polymtl.ca; 5 Department of Nuclear Medicine and Radiobiology, Faculty of Medicine and Health Sciences, Université de Sherbrooke, 12e Avenue Nord, Sherbrooke, QC J1H 5N4, Canada; benoit.paquette@usherbrooke.ca; 6 Clinical Research Center of the Centre Hospitalier Universitaire de l’Université de Sherbrooke, 12e Avenue Nord, Sherbrooke, QC J1H 5N4, Canada; 7 Research Center on Aging, 1036 Rue Belvédère Sud, Sherbrooke, QC J1H 4C4, Canada

**Keywords:** brain cancer, chemoattractant, CXCL, CXCR, glioblastoma multiforme, 3D cell culture systems

## Abstract

Glioblastoma multiforme (GBM) is a grade IV glioma considered the most fatal cancer of the central nervous system (CNS), with less than a 5% survival rate after five years. The tumor heterogeneity, the high infiltrative behavior of its cells, and the blood–brain barrier (BBB) that limits the access of therapeutic drugs to the brain are the main reasons hampering the current standard treatment efficiency. Following the tumor resection, the infiltrative remaining GBM cells, which are resistant to chemotherapy and radiotherapy, can further invade the surrounding brain parenchyma. Consequently, the development of new strategies to treat parenchyma-infiltrating GBM cells, such as vaccines, nanotherapies, and tumor cells traps including drug delivery systems, is required. For example, the chemoattractant CXCL12, by binding to its CXCR4 receptor, activates signaling pathways that play a critical role in tumor progression and invasion, making it an interesting therapeutic target to properly control the direction of GBM cell migration for treatment proposes. Moreover, the interstitial fluid flow (IFF) is also implicated in increasing the GBM cell migration through the activation of the CXCL12-CXCR4 signaling pathway. However, due to its complex and variable nature, the influence of the IFF on the efficiency of drug delivery systems is not well understood yet. Therefore, this review discusses novel drug delivery strategies to overcome the GBM treatment limitations, focusing on chemokines such as CXCL12 as an innovative approach to reverse the migration of infiltrated GBM. Furthermore, recent developments regarding in vitro 3D culture systems aiming to mimic the dynamic peritumoral environment for the optimization of new drug delivery technologies are highlighted.

## 1. Introduction

Malignant tumors of the central nervous system (CNS) are a widely heterogeneous and polygenic disease that constitutes 1.6% of all cancer cases worldwide [1]. The GLOBOCAN cancer tomorrow prediction tool at the Global Cancer Observatory (GCO) (gco.iarc.fr) expects the incidence and mortality rates to increase by more than 41.3% and 46.6%, respectively, by 2040 [2]. In the US, more than 13,000 new patients are diagnosed with Glioblastoma (GBM) every year, 50% of them die within one year, and 90% within three years post-diagnosis [3].

The 2021 WHO fifth edition classified the CNS neoplasms into twelve categories. Within the category of “gliomas, glioneuronal and neuronal tumors”, six large groups of diffuse gliomas are defined: (1) Adults diffuse gliomas, including three types (Grade 2, 3 or 4 Isocitrate Dehydrogenase (IDH)-mutated diffuse astrocytomas, grade 2 or 3 IDH-mutated diffuse astrocytomas, and grade 4 IDH-mutated astrocytoma or Glioblastomas), (2) Pediatric-type diffuse low-grade gliomas, (3) Pediatric-type diffuse high-grade gliomas, (4) Circumscribed astrocytic gliomas, (5) Glioneuronal and neuronal tumors, and (6) Ependymal tumors [4]. Based on the level of the tumor malignancy, grade 1 gliomas can usually be cured by surgical resection, chemotherapy, and radiotherapy. Whereas grade 2 gliomas are highly malignant and can progress to grades 3 and 4, which are more aggressive, invasive, and resistant to the actual standard treatments [5].

GBM accounts for more than 82% of all malignant gliomas [6]. GBM is considered the most aggressive and fatal primary brain tumor, with a worldwide incidence rate of less than 10 per 100,000 [7] and a survival rate of less than 5% after five years post-diagnosis [6]. The incidence of GBM is 1.6 times higher in males compared to females and diagnosed at an older age with a median age of 64 at diagnosis [7,8]. GBM incidence is twice higher in Caucasians compared to Africans and Afro-Americans, and lower in Asians and American Indians [9]. GBM includes “Primary GBM (or de novo)” progressing from low-grade astrocytomas or oligodendrocytomas, which occurs mostly in elderly patients (usually over 60 years old), and accounts for 90% of the GBM cases. On the other hand, “Secondary GBM” accounts for the remaining 10%, generally observed in younger patients (45 years old), and arises from already existing grade I or II gliomas.

**Figure 1:**
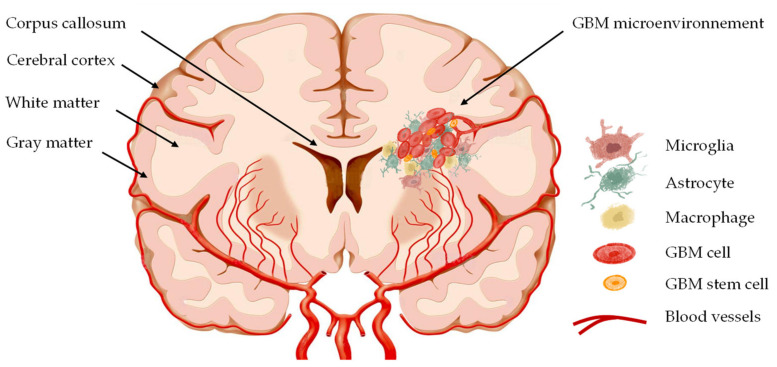
Schematic illustration of GBM.

GBM is characterized by considerable mitotic activity, high vascular proliferation, abundant necrosis, recurrent mutation in IDH in most of its cells, and a high resistance of GBM cells to radio- and chemotherapy. These are the critical factors that make GBM extremely complicated to eradicate. Furthermore, GBM cells have a highly invasive nature and can modulate their microenvironment to disperse and invade the brain parenchyma using the blood vessels and the white matter (Figure 1).

These cells make GBM a disseminated tumor that is impossible to completely resect or cure by the standard treatments that consist of a primary resection surgery, followed by chemotherapy and radiotherapy. Surgery is often powerless due to the depth of the lesion and its location, whereas the radiation tolerance of the brain limits the radiotherapy efficacy. Furthermore, the systemic delivery of chemotherapeutic drugs into the tumor site is limited due to the blood–brain barrier (BBB). Despite its high local aggressiveness, GBM rarely leaves the brain. The estimated incidence of metastasis is seldom up to 0.5% [10], but when it occurs, metastatic cells target mostly the lungs, lymph nodes, liver, bones, and pancreas [10,11,12,13,14]. All these data illustrate the urgent need for novel and innovative alternative treatments for GBM. Here, we first review some of the actual treatments used for GBM. Then, we discuss the roles of chemokines and the interstitial fluid flow in GBM cell invasion (particularly the chemokine CXCL12 and its receptor CXCR4). Finally, we review the recent innovations in drug delivery technology for GBM treatment. We focus on drug delivery devices in GBM and their systemic or local administration, and we finally discuss the strategy we are developing, which is a gliotrap based on the controlled delivery of a chemoattractant. The gliotrap consists of chemoattracting GBM cells with the aim to inverse their migration direction towards being well-confined meaning they can be more efficiently irradiated.

## 2. GBM Traditional Treatments

The high mobility of invading cells, the cellular heterogeneity within GBM clusters, and the ability of GBM cells to transit between proliferative and non-proliferative phases are the main factors responsible for the failure of GBM standard treatment [15,16,17,18].

### 2.1. Surgical Resection

Surgical resection is considered the most important component in the treatment of GBM. Surgery is mainly used for reducing tumor burden, reversing neurological deficits, and the local introduction of therapeutic agents [19]. The feasibility of surgery depends basically on the localization and the size of the tumor. Tumors located in sites such as the eloquent cortex, brainstem, or basal ganglia do not allow surgery, and patients generally have the worst prognosis [20]. However, a successful identification of the tumor margins and its location are still the major challenges that face a total excision. Imaging is the key tool for GBM resection to guide biopsies and identify the tumor margins [19,21,22]. Imaging techniques such as functional magnetic resonance imaging (MRI), computed tomography (CT), and ultrasound now make it possible to process a more aggressive surgical resection with fewer side effects in patients [23]. Even if the surgical resection can remove most of the tumor bulk, the main problem remaining is that a subset of GBM cells at the time of treatment will have already migrated and spread beyond the visible boundary of the main mass [24]. Due to this, radiotherapy postsurgical treatment is usually necessary to prevent recurrence.

### 2.2. Radiotherapy

In the late 1970s, radiation therapy after surgery was shown to improve survival in patients with GBM and has since become progressively optimized for focused applications [25]. After the resection of the tumor, the total radiotherapy dose administrated is typically 60 Gy delivered through 30 fractions of 2 Gy per day (5/7 days) for 6 weeks with intervals [26,27]. In addition to the tumor, a 20–25 mm margin is irradiated in order to kill the infiltrated GBM cells [28,29]. The use of radiotherapy has revealed several limitations and different side effects including cognitive impairment, radiological necrosis, radiation-induced neuronal damage, radio-resistance of some tumors, and reduced proliferation of normal cells caused by DNA damage [30]. However, the other potential radiotherapies-based strategies that have been explored, such as treatments with conformal radiation therapy, stereotactic radiosurgery [31], intensity-modulated radiation therapy [26], and boron neutron capture therapy [32], have shown less toxicity and less exposure in comparison with normal tissues [33]. Conformal radiation therapy, for example, is designed to target the residual GBM cells while maintaining the healthy brain tissue and minimizing the cognitive side effects [25]. Stereotaxic radiosurgery (SRS) consists of delivering X-ray energy in a few sessions (<5) over 6 to 8 weeks [34,35]. Although, clinical neurological complications such as motor deficit, visual deficit, cognitive deficit, sensory deficit, headache, and others are often reported [34]. Nevertheless, the best results for GBM treatment are obtained when radiotherapy (60 Gy) is usually combined with concurrent daily temozolomide (TMZ) chemotherapy [27].

### 2.3. Chemotherapy

Chemotherapy was found to be beneficial for improving patient survival, but the systemic administration of drugs is known to be very limited due to the presence of the BBB [36]. The BBB is the boundary between the circulatory system and the brain extracellular space and is mainly composed of endothelial cells that make tight junctions along the wall of blood vessels [37]. After surgery, the standard treatment regimen includes 6 weeks of concomitant TMZ (75  mg/m^2^ of body-surface area per day for each week) and radiation followed by the administration of adjuvant TMZ (150–200  mg/m^2^) for 5 days every 28 days on six cycles [38]. TMZ, which is a small alkylating agent that methylates the purine bases of the DNA, was first approved by the Food and Drugs Administration (FDA) and used to treat brain tumors in 1993 [39]. So far, its use has shown a significantly better overall survival in treated patients [40]. TMZ reduces the activity of the DNA repair enzyme O 6 methylguanine-DNA methyltransferase (MGMT) to promote GBM tumor cells death. MGMT gene methylation is correlated with a positive prognostic of TMZ [41]. Generally, the major side effect of TMZ is hematologic toxicity [42]. Several agents which alkylate DNA base pairs have been explored in the context of GBM treatments [43]. Carmustine (BCMU), for example, induces a crosslink between the guanine and cytosine. It has been approved by the FDA to treat high grade gliomas (3 and 4) [44]. In addition, in 2014, the FDA approved the use of Lomustine (CCNU) in patients with GBM, following surgery and radiotherapy [45]. Lomustine is a small lipophobic alkylating anti-tumor reagent able to cross the BBB [38].

However, several studies have shown that combining therapies were more effective than using a single approach [46,47]. In 2005, a phase 3 clinical trial conducted by the European Organization for Research and Treatment of Cancer (EORTC) and the Clinical Trials Group of the National Cancer Institute of Canada (NCIC CTG) showed that patient survival significantly increases when treated with TMZ in combination with radiotherapy and complete surgical resection [48]. Unfortunately, due to its various genetic–epigenetic alterations and its high heterogeneity, the standard treatments for GBM remain mostly ineffective to completely heal patients. Therefore, understanding the pathways used by GBM cells to migrate, the molecules that regulate it, and the different mechanisms responsible for the cancer cells extensive invasiveness and migration is critical to develop therapeutic approaches to treat GBM patients.

## 3. CXCL12–CXCR4 in Glioblastoma Invasion

### 3.1. The Pathways of GBM Cell Invasion

**Figure 2:**
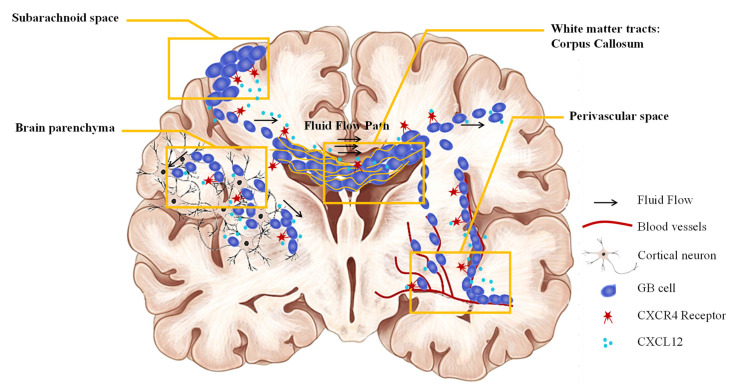
Routes of GBM cells to metastasize in the brain (Adapted from deGooijer et al., 2018 [56]).

Despite several studies and current therapies, GBM treatment remains a challenging task in clinical oncology because of its highly invasive properties, GBM cells being capable of invading local and distant brain tissues. In 1938, Dr. Scherer described the migration of GBM cells following four pathways (Figure 2): (a) within the brain parenchyma while interacting with cells and neuronal extensions; (b) along the blood vessels; (c) following the fibrous pathways of the white matter tracts (in the corpus callosum); and (d) along the subarachnoid space in continuity with the ventricles [17].

The invasion of the parenchyma by the GBM cells is a complex process based on different interactions between the tumor cells and their microenvironment [49,50,51]. The GBM cells have the ability to adapt their microenvironment by modifying the brain extracellular matrix (ECM), which is a complex mixture of molecules such as glycoproteins—fibronectin, laminins, tenascins (TN) as well as glycosaminoglycans (hyaluronic acid, HA), and proteoglycans that contribute to GBM angiogenesis, invasion, and therapeutic resistance [52]. GBM cells synthesize some ECM components such as HA, TN-C, and fibronectin, and express their cell receptors, such as specific integrins [53] and CD44 (receptor of hyaluronic acid (HA)), which leads to cell adhesion and migration [54].

Proteases, such as plasminogen/plasmin system, and cathepsins, that are divided into six groups including gelatinases, collagenases, stromelysins, matrilysins, and metalloproteinases (MMP), are other essential components of the ECM playing an important role in its remodeling during tumor cell migration [53]. For example, the ECM changes its conformation through the action of MMPs, which can be expressed by brain cells and favor GBM cell migration [55]. Through signals mediated by integrins, the ECM also allows GBM cells to develop protrusions and eventually remodel their cytoskeleton to create new connections and influence their migration [53].

The name “integrin” was first proposed by the research group of Hynes in 1986 for a protein complex linking the ECM to the actin-based cytoskeleton [57]. Integrins are the major receptors of the ECM proteins [58], composed of α and β transmembrane heterodimer subunits combined to make 24 different integrins [59]. Currently, 18 α subunits and 8 β subunits have been identified, and each combination determines the functional specificity of the receptors [60]. Based on affinities to ligands, integrins can be classified into four groups: the (a) Collagen receptors group, (b) Laminin receptors group, (c) Leukocyte-specific receptors group, and (d) the group of receptors that recognizes the sequence of three amino-acids RGD (Arginine–Glycine–Aspartate) [61].

**Figure 3:**
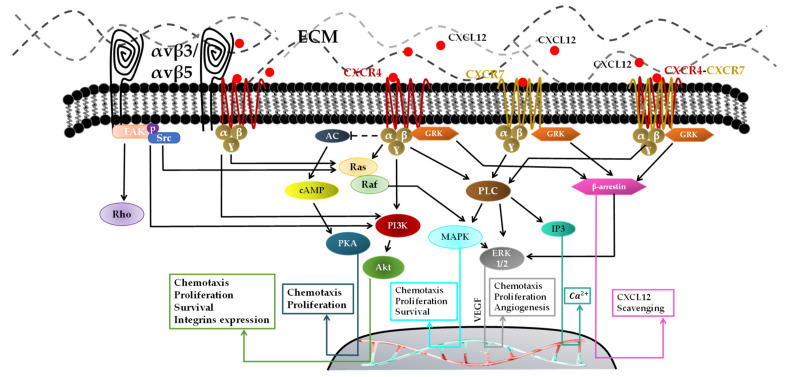
Schematic representation of different signaling pathways activated by CXCL12/CXCR4/CXCR7 axis and integrins. FAK: focal adhesion kinase; p: phosphorylation; Src: proto-oncogene tyrosine-protein kinase; cAMP: cyclic AMP, adenosine 3′,5′-cyclic monophosphate; AC: adenylate cyclase; PKA: protein kinase A; PI3K: phosphoinositide 3 kinase; Akt: protein kinase B; MAPK: mitogen-activated protein kinase; PLC: phospholipase C; ERK: extracellular signal-regulated kinase; IP3: inositol trisphosphate; GRK: G-protein coupled receptor kinase; ECM: extracellular matrix; Ca^2+^: Calcium; CXCL12: CXC chemokine ligand 12; CXCR4: receptor CXC type 4; CXCR7: receptor CXC type 7; αβɣ: G protein complex; αvβ3: integrin alpha V and integrin beta 3; αvβ5: integrin alpha V and integrin beta 5; VEGF: vascular endothelial growth factor.

Integrins play different roles, such as cell adhesion, intracellular transduction, and the regulation of several signaling pathways (Figure 3) [62]. They are also involved in the survival, proliferation, and migration of cancer cells [63]. The integrin-mediated signaling pathway maintains homeostasis, but is dysregulated in tumors and generally associated with the invasive phenotype of GBM cells [64]. For example, RGD-binding integrins include a series of αv dimers, such as αvβ3 and αvβ5, highly expressed in GBM cells in which ligands are ECM proteins, such as fibrinogen (one of the markers of inflammation in brain injury [65]), fibronectin, and TN [66,67]. Types of TN, such as TN-C, can promote cell migration, angiogenesis, and proliferation. TN-C is highly associated with blood vessels which enable glioma cells to invade into other regions of the brain either by binding to integrins to affect the cell directly or by binding to ECM molecules and affecting the cell indirectly [54].

### 3.2. CXCL12–CXCR4 Axis in GBM Cell Migration

Different studies have shown that integrins such as αvβ3, αvβ5, α3β1, α5β1, α6β1, and α9β1 are associated with the invasive phenotype of GBM cells [68,69,70,71]. Blandin et al. demonstrated that αv integrin allows the dissemination of GBM cells in the presence of a rich microenvironment of fibronectin, while its absence reinforces cells adhesion [72]. The stimulation of αv integrins subunits by ECM proteins leads to the activation of different signaling pathways such as phosphatidylinositol-3-kinase (PI3K) and protein kinase B (AKT) pathways, for which several studies have proven their induction of GBM cell migration [64,72,73]. Rat sarcoma virus (RAS) and extracellular signal-regulated kinase (ERK) pathways are also activated via the increased activity of MMP-2 and MMP-9 [74,75]. In conclusion: (a) Integrins’ activation via ECM proteins leads to changes in the GBM cell cytoskeleton and the activation of gene transcription for cell adhesion, migration, and invasion [76,77,78,79]. (b) The ECM has become a target for many researchers due to the important role it plays in GBM progression. For more information about the emerging therapeutic approaches for GBM that target ECM, see Mohiuddin et al., 2021 [54].

However, in addition to the ECM, the GBM microenvironment is constituted from several other components, such as fluids, molecules such as chemokines that play a crucial role in the migration of GBM cells, and others, such as neurons, astrocytes, and immune cells [55].

### 3.3. Chemokines Implicated in GBM Cell Invasion

Chemokines are a family of small (8–10 kDa) secretory proteins whose name comes from their ability to be “chemoattractant cytokines” [80]. Chemokines are known to allow the migration of various cell types such as leukocytes, fibroblasts, and both normal and malignant epithelial cells [80]. Chemokines are divided into four subfamilies based on the positioning of their cysteine residues from the amino-terminus side: CXC, CC, C, and CX3C [81]. By binding to their receptors, chemokines activate various intracellular signaling pathways and play a crucial role in the regulation of many biological processes such as angiogenesis, immune response, hematopoiesis, chemotaxis, as well as cell proliferation, migration, apoptosis, and others [82].

Forty-seven chemokines and twenty chemokine receptors have been identified in humans so far [83]. Chemokine receptors such as CXCR are G-protein-coupled receptors (GPCR) that include a central common core made of seven transmembrane helices (TM-1 to -7). Helices are connected by three intracellular and three extracellular loops, also an N-terminal extracellular domain and C-terminal intracellular domain that is specific to every protein receptor [84]. A GPCR can bind to its ligand either by homodimerization (one receptor for one G-protein) or by heterodimerization [84]. Once stimulated, GPCR modifies its three-dimensional conformation; guanosine triphosphate (GTP) and guanosine diphosphate (GDP) exchange is activated, which leads to the dissociation of the GTP-bound α-subunit and βγ-dimer [85]. Gαi induces the inhibition of adenylate cyclase, which prevents cyclic adenosine monophosphate (cAMP) production and activates Src-like tyrosine kinases, while Gαq activates phospholipase C (PLC). This enzyme hydrolyzes the phosphatidylinositol 4,5-bisphosphate into diacylglycerol and inositol 1,4,5 trisphosphate (IP3), allowing the release of intracellular Ca^2+^ from the endoplasmic reticulum (Figure 3) [86,87]. On the other side, the Gβγ dimer activates numerous signaling pathways involved in the migration of GBM cells, in particular, PI3K, Akt, ERK1/2, mitogen-activated protein kinase (MAPK), and serine/threonine-specific kinases (Raf) pathways [88,89,90]. CXCL12 can also induce the phosphorylation of CXCR4 by GPCR kinase (GRK), leading to its subsequent interaction with β-arrestin. Certainly, for its formation and progression, GBM cells engage different chemokines such as CXCL8, CXCL16, CX3CL, CCL5, CXCL12, and their receptors CXCR1-2, CXCR6, CX3CR1, CCR5, CXCR4-7, respectively, for a chemokine network communication mechanism to maintain and increase the tumor malignancy (Table 1).

Among several chemokines and their receptors, CXC chemokine ligand 12 (CXCL12) and its two GPCR receptors, CXC type 4 (CXCR4) and type 7 (CXCR7), are mainly involved in the migration and the spreading of GBM cells into distant tissues.

CXCL12 plays an important role in the regulation of different processes such as neuronal development and stem cell motility [91]. CXCL12 has been found in the white matter tracts, blood vessels, and subpital regions, suggesting chemotactic direction cues for GBM cell invasion [99]. Its high expression in some areas, such as white matter tracts, can also attract the spread of the cells toward those areas, causing invasion [14]. CXCR7 has been recently identified as a second receptor for CXCL12, and its expression has been shown to be increased in many tumor cell lines, including gliomas cells [100]. It has a 10-fold higher affinity to CXCL12 than CXCR4 [101]. Nonetheless, CXCR4 plays a crucial role in GBM invasion [100] by mediating chemotaxis in the brain. Being widely expressed within the human body, the CXCR4 upregulated state has been reported in over 20 types of human malignant tumors such as breast, prostate, lung, ovarian, and brain cancer [102,103,104]. The receptor is also overexpressed in GBM and is considered as a hallmark of the tumor aggressiveness [105,106]. The CXCL12/CXCR4 axis has gained increased focus during the recent decade [107] and has been extensively studied in brain tumors (GBM, astrocytoma, medulloblastoma, oligodendroglioma, and oligodendroastrocytoma) [108] due to its key role in the communication of tumor cells with their microenvironment [109]. The axis is implicated in GBM immunosuppression, chemotherapy and radiotherapy resistance [107,110], cellular reprogramming, and ECM remodeling [107]. CXCL12 acts primarily via two mechanisms in cancer: (a) through an autocrine effect that promotes cancer cell growth, invasion, and angiogenesis, and (b) indirectly by recruiting cancer cells expressing CXCR4 into regions or organs containing CXCL12 to initiate metastasis [111].

Both in vitro and in vivo studies proved that CXCL12 promotes GBM growth and cell migration, and inhibits apoptosis through the activation of various signal transductions [112]. The CXCL12–CXCR4–CXCR7 axis has been extensively studied over recent years [91]. It acts via three mechanisms (Figure 3):

(a) Through CXCR7, which has been known for decades and has an inability to activate the G-protein complex [113]. Lately, it has been proved in vitro that CXCR7 activation by CXCL12 is mediated via G-protein and β-arrestin and increases the intracellular calcium concentration. β-arrestin has four isoforms and plays a key role in GPCR signal transduction [114]. It activates numerous intracellular signaling pathways such as MAPK-ERK1/2 pathways for cell proliferation and migration [115]. An in vivo study demonstrated that the inhibition of CXCR7 after irradiation prolonged survival and blocked tumor recurrence of intracranial U251 GBM in nude mice [116]. Yang Liu et al. showed that knocking down CXCR7 in GBM cells (U251MG and U373MG) using siRNA to block ERK1/2 in response to CXCL12 decreases cell proliferation, invasion, and migration [93].
(b) CXCR7 can heterodimerize with CXCR4 in response to CXCL12, which modulates CXCR4-mediated G-protein and β-arrestin signaling pathways such as MAPK-ERK1/2 inducing cell migration [92].
(c) One CXCL12 binds to its receptor CXCR4 [117,118], the tertiary structure of CXCR4 changes to activate the G-protein through its intracellular component. Multiple signals are activated via GRK, such as phospholipase C (PLC), PI3K, and MAPK/ERK, for vascular endothelial growth factor (VEGF) production, which is mainly responsible for recruiting new vessels for GBM neovascularizations [119]. The activation of the PI3K pathway followed by the activation of Akt contributes to the CXCL12/CXCR4-induced survival, invasion and proliferation. CXCL12–CXCR4-mediated migration is reported to be regulated by the PI3K and MAPK/ERK pathways [119].

Overall, targeting the CXCL12–CXCR4–CXCR7 axis to develop new therapeutic approaches in GBM treatment is required. AMD3100, Peptide R, CPZ1344, and AMD3465 are inhibitors for CXCR4 that reduce tumor growth and inhibit GBM cell invasiveness and migration [120]. The most extensively studied, AMD3100, also known as plerixafor (Mozobil^®^), is an FDA-licensed CXCR4 antagonist that was approved in 2008 [121]. In addition, AMD3100 reduces the chemotaxis, survival, and proliferation of glioma cell lines [120]. For instance, Cornelison et al. proved that AMD3100 can inhibit CXCR4-dependant GBM invasion induced by convective flow forces within the tumor tissue [106].

Furthermore, it has been also demonstrated that CXCL12 and CXCR4 induce the activation of integrins which suggests a cooperation between the CXCL12–CXCR4 axis and the integrins in mediating cancer cell behavior, such as adhesion and survival [122]. For example, the adhesion of prostate tumor cells to the endothelium or proteins of the ECM is transmitted via CXCL12-induced signals depending on CXCR4. The binding of CXCL12 to its receptor leads to an upregulated expression of α5 and β3 integrin subunits. In contrast, the level of α2, β1, β4, and αv integrin subunits remains unchanged in CXCL12-treated cells. This provides evidence of existing links between CXCR4 expression, CXCL12, and α5 and β3 integrin subunits [118,123,124]. As the CXCL12–CXCR4 axis is the potent driver for GBM invasion under static conditions, studies have shown that cell invasion and migration may be enhanced by another factor, interstitial fluid flow [99,106,125].

## 4. Interstitial Fluid Flow in Glioblastoma

### 4.1. Cerebral Fluids

Within the brain, many processes occur to regulate different mechanisms with the aim of maintaining homeostasis. Cell communication, environment interactions, and chemical gradients are necessary to ensure the brain function. On another scale, biophysical forces such as fluid flow act by ensuring the creation of the chemokine gradient in neonatal and adult development, and by recruiting immune cells into target sites [69]. Brain tissues are composed of three compartments: neural cells, the vascular system, and the interstitial space (IS) [126]. The latter occupies 15% to 20% of the total brain volume and is composed mainly of interstitial fluid (IF) and the extracellular matrix (ECM) [126]. IF is one of the most important axes studied because of its behavioral changes in pathological diseases such as CNS cancers and Alzheimer’s disease [127]. IF surrounds every cell in the brain, allowing it to be the carrier for proteins and different molecules to and from cells [128]. The interstitial fluid flow (IFF) can be defined as the movement of fluid between cells in the interstitial space. It is mainly composed of water, ions, and gaseous and organic molecules (O_2_, CO_2_, hormones) [128]. Chary and Jain measured the interstitial flow velocity using fluorescence intensity after bleaching on a rabbit ear, which was found to be 6 × 10^−5^ cm/s. In GBM, the IFF develops due to the high interstitial pressure between the tumor and the healthy tissue [129]. Besides the hypotheses that the ECM and cytokines increase GBM cells’ invasion, the IFF has been proved to enhance GBM cells’ migration as well.

### 4.2. Interstitial Fluid Flow and CXCL12 in the Migration of GBM

The IFF plays a critical role in the GBM cell invasion of the brain parenchyma [99,106,125] by enhancing CXCL12 secretion along white matter tracts, blood vessels, and subpial regions [130]. The fluid flow in the brain also follows the same pathways, providing the information that the fluid flow, CXCL12 gradients, and white matter tracts may be interrelated. The IFF stimulates GBM cell migration mainly by mediating two mechanisms: (a) Autologous chemotaxis, in which the GBM cell stimulates its migration via a self-secreted chemokines mechanism. The secreted chemokine allows the cell to be transported following the direction of the flow, creating a gradient around individual cells [106]. (b) Mechanotransduction, which occurs when a cell detects mechanical changes in its environment, leading to the activation of different signaling pathways mediating the migration [131].

For example, the fluid flow was found to enhance glioma cell migration via a CXCL12–CXCR4 signaling-dependent mechanism in both 3D in vitro models using invasive cells (RT2, rat astrocytoma; U87MG, human glioblastoma; C6, rat astrocytoma) in a HA/collagen I matrix and in vivo models such as rats [99]. The response of these invasive cells to the fluid flow (velocity of 0.7 µm/s) was prevented by blocking CXCR4, either by adding its inhibitor AMD3100 (Mozobil^®^, Plerixafor) or CXCL12 at 100 nM (uniform CXCL12 treatment) to abrogate any chemoattractant gradient [99]. Nonetheless, blocking CXCR4 did not stop the GBM cell invasion entirely under static or flow conditions, suggesting that the cells are capable of producing their CXCL12 to migrate [99]. Cornelison et al. used an in vitro 3D tissue culture insert model to show that GL261 cells can migrate under static conditions, which can be significantly enhanced after flow application. Furthermore, they used convection enhanced delivery (CED), a technique known to enhance the local perfusion of chemotherapeutic agents in the treatment of GBM, to highlight the invasion effects of convective forces on glioma cell invasion into the surrounding brain parenchyma in an in vivo mice model [106]. The flow effect can be mitigated by blocking the CXCL12 gradient or its receptor using uniform CXCL12 and AMD3100, respectively, [106].

However, Kingsmore et al., using G2, G34, G62, and G528 GBM stem cell lines, proved that the response to the fluid flow is heterogenous and can vary from one cell line to another, both in vitro and in vivo [125]. The invasion of G34, G62, and G528 GBM stem cell lines increased in response to the interstitial fluid flow as compared to the static conditions, unlike the G2 invasion that was not increased, which was in accordance with their in vivo results. Using antibodies against CXCR4 and CXCL12, the authors also found that G34 and G528 cells respond to the flow through CXCR4/CXCL12 chemotaxis. Uniform CXCL12 also decreased the flow-stimulated invasion in both G34 and G528 GBM stem cells, confirming the crucial role played by the chemoattractant for flow-responsive invasion in these cells [125]. Interestingly, flow-stimulated invasion in G34 cells also depended on CD44, a receptor of HA mediating mechanotransduction [125].

In contrast, Qazi et al., by culturing U87 human glioma, rat CNS-1 glioma, and U251 human glioma cell lines in 3D modified Boyden chambers containing collagen type I in a dynamic microenvironment with various flow velocities (0.8–3 µm/s), found that the migration rate of the cells diminished after exposure to the flow. This decrease of migratory activity by flow was dependent on MMP-1 and MMP-2 for U87 and CNS-1 cells, respectively. In contrast, the migration of U251 cells was not affected by the shear stress. However, adding chemoattractant TGF-α with the flow enhanced the U87, CNS-1, as well as U251 cell migratory activity by 89%, 566%, and 101%, respectively, compared to controls [132]. All this indicates that CXCR4/CXCL12 mechanisms can have different impacts on the GBM cell population depending on their heterogeneity, which may contribute to different invasion behavioral responses to fluid flow. However, since studying GBM cell migration in vivo is currently limited, bioengineers focus on the development of in vitro models that mimic different elements of the in vivo microenvironment.

## 5. Models to Study Glioblastoma Cells Migration

### 5.1. Two-Dimensional Models for Glioblastoma Studies

Several studies performed in two/three-dimensional in vitro and in vivo models have proved that GBM cells invade either by an amoeboid or mesenchymal manner [133,134]. Two-dimensional models are simple to use, but have proved to have several limitations in terms of mimicking the natural microenvironment of the parenchyma. Scratch assays are the commonly used technique. They allow the study of migration in favor of invasion, but do not allow the distinction between the proliferative and invasive behavior of the cells. Transwell assays, on the other hand, give more information but produce a wider range of results [135]. However, these kinds of tests fail to recapitulate the real microenvironment of the brain [136] (Table 2).

### 5.2. Three-Dimensional Models for Glioblastoma Studies

Three-dimensional assays are a step forward to investigate cell invasion as they mimic the cellular microenvironment more closely. Boyden chambers are the most used. They consist in evaluating the cell invasion through a porous insert that can be coated with different matrices, such as the mouse sarcoma-derived matrigel or collagen-based matrices [56]. The matrices are static, so the addition of chemo-attractants in the lower compartment is usually required in most cases [142]. Microfluidic co-culture platforms are 3D in vitro models made of a gel scaffold bound by two channels in which cells can be seeded. This allows the study of the impact of intracellular interactions on GBM invasion (see Table 2) [56,143]. Cerebral organoids on another side have been recently developed to culture tumor cells retaining part of the original brain morphology [144]. Models such as organotypic brain slice cultures are ex vivo models that have emerged at the beginning of this century to mimic in vivo orthotopic xenograft invasion models [56]. These models allow GBM cells to express their invasive characteristics in a physiological microenvironment as well as their interactions with the ECM [52,145].

## 6. Innovative Treatments for Glioblastoma

**Figure 4:**
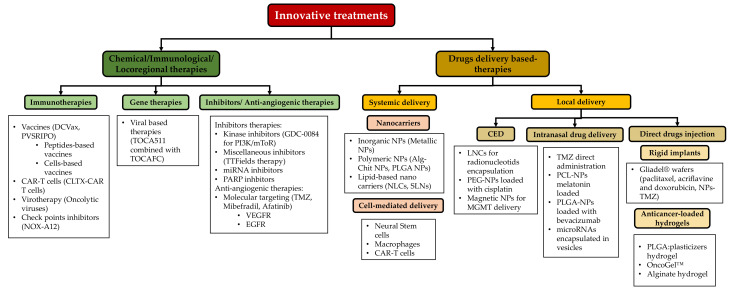
Summary of some different innovative treatments for GBM. DCVax: dendritic cells vaccine; PVSPIRO: recombinant nonpathogenic polio-rhinovirus chimera; CAR-T: chimeric antigen receptor T cell; CLTX-CAR T: chlorotoxin-chimeric antigen receptor T cell; NOX-A12: olaptesed pegol; TOCA511: vocimagene amiretrorepvec; TOCAFC: toca 511 (vocimagene amiretrorepvec) + FC (5-fluorouracil); GDC-0084: paxalisib; TTFields: tumor-treating fields; PARP: poly (ADP-ribose) polymerase; Alg: alginate; Chit: chitosan; PLGA: poly D,L-lactic-co-glycolic acid; NLCs: nanostructured Lipid Carriers; SLNs: solid lipid nanoparticles; LNCs: lipid nanocapsules; PEG: polyethylene glycol; PCL: polycaprolactone.

Despite the advanced clinical therapeutic approaches, the combination of classical treatments yet fails to cure GBM due to tumor recurrence and metastasis. Therefore, varied applications of biotechnology and many innovative approaches such as immunotherapies, gene therapies, and drug delivery-based therapies have been explored (Figure 4) and will be briefly discussed. In this review, we will focus mainly on describing the different drug delivery therapies used in the development of new GBM treatments.

### 6.1. Some Innovative Treatments in Glioblastoma

#### 6.1.1. Gene Therapies

Gene therapy consists of integrating a functional version of a gene instead of the defective one. There is no gene therapy clinically approved at the moment, but various clinical trials with encouraging results are ongoing [146,147]. Many strategies have been used in the context of gene delivery, such as antiangiogenic gene therapy and nano-technology-based gene therapy in the past twenty years [148].

Some of the antiangiogenic gene therapies have been successful to suppress tumor angiogenesis and growth [148]. Angiogenic gene therapy strategies consist mostly in disrupting the normal function of VEGF [149]. For example, ofranergene obadenovec (VB-111) is a genetically modified non-replicating adenovirus type 5 containing a specific promoter and a transgene encoding for a chimeric death receptor (proapoptotic Fas-TNFR1 chimeric protein) [150,151]. Cloughesy et al. have performed a phase III clinical trial (256 patients at 57 sites) studying the effect of VB-111 administration with and without bevacizumab, a humanized monoclonal antibody for VEGF-A, approved for use in recurrent GBM in 2009 [152,153]. The patients treated with VB-111 showed apoptotic cell areas in the tumor with an increase in the number of infiltrated CD8 and CD4 lymphocytes. However, the administration of VB-111 and bevacizumab did not improve the outcomes in recurrent GBM [152]. Some other studies hypothesized that combining TMZ chemotherapy with gene therapy may be beneficial for a synergistic effect against GBM and that the decrease of the MGMT level with gene therapy can overcome TMZ resistance and enhance GBM cell death [154,155,156]. For example, Przystal et al. introduced a unique prokaryotic viral-based approach combined to TMZ to target tumors [155]. Using a M13 bacteriophage that specifically infects bacterial cells, the single-stranded genome of human adeno-associated virus (AAV) was inserted in and the phage capsid was engineered to display the CDCRGDCFC (RGD4C) ligand that binds to αvβ3 integrins [155]. Once bonded to these integrins, the RGD4C/AAV-Phage (AAVP) viral particles penetrate the GBM cells, releasing the AAV genome that expresses a tumor-specific gene from the *Grp78* promoter. Przystal et al. first verified that TMZ increases the expression of the endogenous Grp78 protein in human GBM cells (LN229, U87, and SNB19 cells) in a dose-dependent manner. They also showed that RGD4C/AAVP-*Grp78* gene expression is strongly increased by TMZ. Interestingly, the combination of the TMZ with the RGD4C/AAVP-Grp78-HSVtk mutant SR39 encoding the Herpes simplex virus type I thymidine kinase in the presence of ganciclovir (GCV) induced strong tumor cell killing both in vitro and in vivo (mice with established intracranial U87 tumors) [155].

mRNA and siRNA in GBM Gene Therapy:

mRNA-based gene therapy is an efficient gene transfection tool that emerged to adapt with the high heterogeneity and diffusing invasiveness nature of the GBM. Xiangjun et al. explored a new therapeutic strategy using an in vitro synthesized mRNA encoding for (a) a phosphatase and tensin homolog on chromosome ten (PTEN) that can induce apoptosis or (b) a tumor-necrosis-factor-related apoptosis-inducing ligand (TRAIL)- in tumor cells derived from -*PTEN*-deletion patients [157]. They confirmed that a low survival rate is observed in GBM patients who have a high frequency mutation of PTEN. Using patient-derived primary GBM stem cells with *PTEN* alteration and a Denver Brain Tumor Research Group (DBTRG)-cell-derived xenograft to detect the cytotoxicity of mRNA in vitro and tumor suppression in vivo, they showed that the combined treatment of PTEN-mRNA and TRAIL-mRNA significantly reduced the growth of both the GBM cells and tumor. The tumor growth is suppressed after two months compared with the control PBS (96.4%) and single mRNA group (PTEN-mRNA (89.7%) or TRAIL-mRNA (84.5%)) [157]. mRNAs can also be encapsulated into nanocarriers such as liposomes and nanoparticles (NP) to overcome the natural barriers and protect them from degradation [158]. The delivery routes and the carrier forms of mRNA depend on the patients glioma grade, stage, surgery, and chemotherapy history [158]. To our knowledge, no clinical trial of mRNA-based GBM therapy has been completed, and has not been widely adopted in treating GBM yet.

On another side, small interfering RNA (siRNA) are known by their ability to silence the genes responsible for cancer progression by targeting tumor-promoting factors, such as VEGF and EGFR [159,160]. Since GBM cells are resistant to anti-tumor drugs, the use of siRNA in combination with chemotherapy could be beneficial to enhancing the treatment efficiency [161]. For example, the combination of resveratrol (RES) and heat shock protein 27-knockdown using siRNA (Hsp27) was tested to treat the disease [162]. Hsp27 is a tumor-promoting factor in GBM implicated in ECM remodeling and cell survival. RES at 15 µM decreases the Hsp27 protein level in a similar way than quercetin, a well-known Hsp27 inhibitor (47% and 41%, respectively). However, combining RES at 15 µM with Hsp27 siRNA induces a decrease in the level of Hsp27 by 93.4% in transfected human U87 MG cells [162]. This combined treatment increases the caspase-3 activity by 101% and induces GBM cell apoptosis. [162]. This study proves that the use of Hsp27-siRNA combined with an anti-tumor agent could be beneficial to induce apoptosis in GBM cells. However, the BBB, the degradation by RNAse enzymes, and reaching the tumor site are the main challenges preventing the efficiency of siRNA therapy [163]. Under these conditions, nanocarriers can promote a targeted delivery of siRNA and protect them against degradation at the same time. A wide range of siRNA-loaded nanocarriers have been tested in GBM therapy [164]. For instance, loaded MGMT-siRNA liposomes have been tested in GBM treatment resulting in MGMT downregulation, DNA repair induction, and decreased drug efflux capacities responsible for increasing GBM cell sensitivity to TMZ [164]. In another study, RGD-functionalized pH-responsive polyamidoamine (PAMAM) dendrimers were investigated for delivery of both c-myc siRNA anddoxorubicin (DOX)-loaded Se NP in GBM therapy [165]. The RGD functionalization of PAMAM enhances the uptake of siRNA dendrimers by cancer cells [165]. The nanocarriers were able to penetrate a BBB model in vitro, developed to deliver the drug and enhance the antitumor activity [165]. Moreover, chitosan lipidic nanocapsules were used for galectin-1 and EGFR-siRNAs’ delivery in nude mice-bearing orthotopic U87 MG GBM cells [166]. The mean survival time increased in the mice treated 14 days after tumor implantation with both anti-EGFR and anti-Galectin-1 siRNAs plus TMZ (39 days), in comparison to untreated mice (32 days), or EGFR siRNA plus TMZ or anti-Galectin-1 siRNA plus TMZ (34 days), representing a promising strategy to induce anti-tumor effects in GBM [166]. Furthermore, CXCR4-targeted peptide carriers for VEGF-siRNA delivery were tested in GBM therapy [167]. The peptide carriers were able to condense and protect siRNA from RNAse degradation and induced a 2–6-fold decrease in VEGF expression in the cells, indicating that the surface modification of the nanocarriers can improve their specificity towards GBM cells [167]. However, more studies to develop safe and well-tolerated nanocarriers for siRNA delivery are needed. Another therapy that appeared to be very powerful and hopeful is immunotherapy, in which drug delivery systems are extensively used.

#### 6.1.2. Immunotherapies

After the success achieved for treating various other cancers, immunotherapies have been considerably investigated to translate the same achievements in GBM treatment. Immunotherapy consists of harnessing the immune system to eradicate the tumor cells and is quite efficient in the treatment of high mutational burden tumors. Since GBM has a low tumor mutational burden and an immunosuppressive environment, various strategies have been explored to boost host immunity against GBM [168].

Among them, immune checkpoint inhibitors release the inhibitory brakes of T cells, activating the immune system to induce anti-tumor responses [169]. When the binding to their ligand is inhibited, the checkpoint receptors can promote an effective cell response against GBM [170]. Targeted checkpoint molecules such as Nivolumab (Opdivo^®^), pembrolizumab (Keytruda^®^), durvalumab (Imfinzi^®^), and atezolizumab (Tecentriq^®^) (an anti-programmed death-ligand 1) have been approved to treat several types of cancer and are currently trialed in GBM treatment [171,172]. For example, Gardell et al. used human monocyte-derived macrophages genetically modified for bispecific T cell engager (BiTE) and proinflammatory cytokine IL-12 secretion [173]. BiTE is specific for the mutation of epidermal growth factor receptor variant 3 (EGFRV3), expressed by the GBM cell. The secreted BiTE, by binding to the tumor antigen, was able to activate T cells as well as their proliferation and degranulation, leading to the elimination of the antigen-specific tumor cells in in vitro and in vivo models. BiTE secretion promotes a reduction in the tumor burden [173]. However, the kinetics release of BiTE protein from the cells still needs to be improved which reveal the importance of the platform optimization in the development of therapeutic approaches. Further, chimeric antigen receptors (CAR), that consist of the use of genetically modified T cells to express CAR genes, have been approved by the FDA for the treatment of hematologic malignancies [174]. O’Rourke et al. were the first to use autologous T cells redirected to the EGFRV3 mutation by CARs on human GBM patients [175]. They demonstrated that the use of CART-EGFRV3 (10 patients) is feasible and safe, since no toxicity was observed [175]. Seven patients had surgery after CART-EGFRV3 treatment, which allowed us to gather more information: (a) The trafficking of CART-EGFRV3 cells was observed in the direction of the tumor within the first 2 weeks of treatment. (b) The in-situ studies of the tumor environment also showed an up-regulation of immunosuppressive molecules, such as indoleamine 2,3-dioxygenase 1 (IDO1), programmed cell death ligand 1 (PD-L1) and IL-10, as well as the recruitment of immunosuppressive regulatory T cells expressing FoxP3. Thus, CART therapy for GBM still needs more investigation because of the immunosuppressive tumor microenvironment, cell trafficking, and risks of CNS toxicity [175].

Some approaches based on vaccines are also investigated as a potential adaptive immunotherapy for GBM. An autologous tumor lysate-pulsed dendritic cell vaccine called DCVax^®^-L, produced by Northwest Biotherapeutics, Inc., Bethesda, MD, USA [176], has been used against glioblastoma and appears to be safe. DCVax-L has been approved for GBM treatment in Switzerland, but is still under clinical trials in the United States [177]. Additionally, a phase 3 trial is ongoing to evaluate the long-term effects of the DCVax^®^-L vaccine in patients after surgery and chemoradiotherapy [178].

On another scale of immunotherapies targeting GBM, viral-based therapy involves genes delivery via viral vectors. For example, Desjardins et al. focused on the use of the convection-enhanced intratumoral delivery of the recombinant nonpathogenic polio-rhinovirus chimera (PVSRIPO) which recognizes the neurotropic poliovirus receptor CD155 widely expressed by GBM cells and improves the survival rate of the patients [179]. In summary, immunotherapies demonstrated promising results in terms of feasibility, safety, and even signs of efficacy. The challenges ahead are still numerous, including the optimization of the dosing, the modulation of immunosuppressive tumor microenvironment, the molecular marked heterogeneity of GBM and the understanding of the chemokines network. Thus, drug delivery systems have emerged to overcome some of those limitations.

### 6.2. Drugs Delivery Systems for Glioblastoma Treatments

#### 6.2.1. Systemic Delivery

The BBB is considered as the major hurdle in drug delivery-based therapies because of its low permeability that hampers the passage of anti-cancer agents (ACA). The transport of ACA is achieved via two mechanisms: (a) passive transport (diffusion of water-soluble compounds and lipophilic molecules with a molecular weight less than 500 Da), and (b) active transport (mediated by membrane protein carriers of small molecules) [180]. Therefore, in order to promote the passage of ACA, different strategies have been explored, including small molecules capable of crossing the BBB, chemical modification of ACA, drug-loaded nanocarriers, and cell delivery systems [181].

Cell-Mediated Delivery:

Cell-mediated delivery utilizes cells such as leukocytes and stem cells to carry drug carriers themselves [182]. The strategy had several advantages such as long circulation times, flexible morphology, and cellular signaling [183]. The drugs can be loaded into the cells through biological pathways (endocytosis, ligand-receptor interactions), physical approaches (hypotonic hemolysis, electroporation), or chemical modifications (covalent conjugation onto surface markers, biotinylation, click chemistry) [184].

Macrophages are commonly used because of their particularity to migrate to the tumor side in response to the secretion of cytokines and chemokines [184]. Cell-mediated delivery has been widely explored in GBM. For example, neural stem cells (NSC) have been used to secrete and deliver the proapoptotic protein TRAIL to human intracranial glioma xenografts [185]. The use of high doses of TRAIL in patients can induce issues of toxicity and a danger of excessive antiviral host immune responses; for this reason, the molecule was delivered by NSCs. NSCs were able to migrate to the tumor site and secrete TRAIL, resulting in the apoptosis of the cancer cells without toxicity for the normal brain parenchyma. They also induced a significant reduction in the tumor size [185]. Wang et al. found that monocyte-mediated DOX (also known as Adriamycin^®^) delivery through NPs, with the surface coated with polyglycerol and RGD peptides for GBM treatment, caused tumor cell damage both in vitro and in vivo in mice orthotopic GBM xenografts. [182]. In the same context, Pang et al. loaded NPs into macrophages such as ”Trojan horses” to deliver DOX for GBM treatment [186]. The viability of the cells encapsulating the NPs was not affected and an improvement of the macrophages’ penetration into the core of the spheroids model was observed, which mimics the behavior of the cells in an in vivo model [186]. To conclude, the tumor targeting was enhanced after loading the NPs into the cells, which indicates that macrophages can improve glioma therapy and underline the importance of using NPs [186].

Nanocarriers:

Nanotechnology and nanocarrier-based drug delivery have recently gained remarkable attention due to their characteristics of biosafety, sustained drug release, and enhanced drug bioactivity and BBB penetrability [187]. Based on preparation methods, nanocarriers can be classified into nanocapsules, nanospheres, and NPs that are the mostly used in GBM treatment.

Nanocapsules are small vesicles of 100–200 nm in which hydrophobic drugs are encapsulated in the empty space by a polymer membrane. Polymers such as poly(lactic acid) (PLA) and Poly Lactic-co-Glycolic Acid (PLGA) can be used to prepare these nanomaterials [188]. NPs can be loaded by different therapeutic agents and are characterized by particular properties that allow them to pass the BBB [189] and achieve the tumor site. The particle size, surface charge, hydrophobicity, and coating material are the NPs’ physiochemical properties that play an important role in the targeting process [190]. The size of the NPs is a critical factor for the NPs’ delivery; small size NPs < 200 nm are preferred and suitable for systemic administration and can smoothly reach the leaky blood vessels of the tumor microenvironment [191,192,193]. The shape, stability, and charge of the NPs are also important due to their implication in fluid dynamics and their interaction with cell charge membranes and proteins [194,195]. Based on their composition and characteristics, NPs can be classified into lipidic NPs, organic NPs, and inorganic NPs [196,197,198].

**Figure 5:**
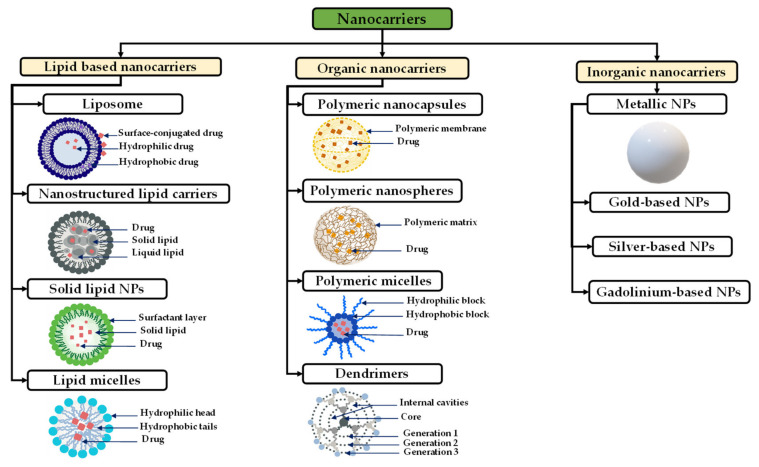
Schematic representation of different types of nanocarriers and their structures used in GBM treatment.

Lipid-based nanocarriers regroup liposomes, nanostructured lipid carriers, solid lipid NPs, and lipid micelles (Figure 5). Liposomes are small vesicles composed of a phospholipidic bilayer that surrounds a water-soluble core similar to the cell membrane [199]. Liposomes are characterized by an easy encapsulation of ACA, an easy preparation, biodegradability, and a favorable biocompatibility [200]. Nanostructured lipid carriers (NLCs) are composed of a matrix with solid and liquid lipids forming an unordered matrix, providing a large space for drug incorporation [201]. Solid lipids NPs (SLNs) are formed from a solid hydrophobic lipid core and have demonstrated a higher stability compared with liposomes [202]. Further, SLNs have the ability to cross the BBB and deliver a wide spectrum of GBM-targeted ACAs, such as large molecules, genes, oligonucleotides, and enzymes [203]. Finally, lipidic micelles are spherical amphiphilic aggregates with a hydrophobic core and a hydrophilic shell in which drugs are loaded in the central core or can be linked to the lipids [201].

Inorganic NPs are generally composed of mineral compounds, such as metals. Among various inorganic NPs: iron oxide NPs are commonly used as contrast agents for MRI, and gold NPs are used to improve photothermal therapy [204,205,206].

For example, Liu et al. prepared magnetic iron-based NPs (MNP) modified by PEG-transferrin (Tf-PEG) and polylysine (PLL) to condense small interference RNA against polo-like kinase I (PLK1) (Tf-PEG-PLL/MNP@siPLK1). The Tf was used to target GBM cells since they express a high amount of transferrin receptors. PLK1 is involved in G2/M transition in the cell cycle and is related to tumor progression and recurrence [207]. They found that Tf-PEG-PLL/MNP@siPLK1 at the dose of 1.6 mg/kg prolonged the survival time of GBM mice, 80% of them being alive at three months. In contrast, a median survival time of about one month was observed in Tf-PEG-PLL/MNP@Scrambled PLK1-treated GBM-mice [207]. On the other hand, Zhu et al. developed ruthenium Tf and aptamer AS1411 co-grafted NPs loaded with [Ru(bpy)2(tip)]2+ (RBT), an antitumor drug for cell apoptosis. Using photodynamic therapy for GBM, they proved that RBT@MRN-SS-Tf/Apt killed glioma cells in vivo and in vitro under laser irradiation, prolonging the median survival rate [208].

Polymeric nanoparticles can be prepared using a different range of materials and are used as carriers for different drugs, such as chemotherapeutic drugs [188,209]. Polymeric nanoparticles are more advantageous over other types of NPs because of their biocompatibility, biodegradability, and the improvement they achieved in the kinetics release as reported in many reviews on the subject [210,211]. Polymeric NPs preparation can be very flexible in terms of composition, structure, and properties [212]. Polymeric NPs can be prepared using: (a) Synthetic polymer NPs such as PLA, poly(ε-caprolactone) (PCL), poly(glycolic acid) (PGA), PLGA, and poly (amino acids) [209,213,214], these polymers are biocompatible and degrade hydrolytically [215]. (b) Natural polymers which are composed of polymers such alginate [216,217], chitosan [216,217], dextran [218], and HA [219].

Polymeric micelles are highly biocompatible amphiphilic nanoparticles capable of delivering different therapeutic agents and characterized by their flexibility in terms of design modification [220]. Polymeric micelles have core–shell-type NPs formed through the self-assembly of block copolymers, and which have a controllable size range of 10–100 nm [221]. Biodegradable polyesters such as PCL, poly (D,L-lactide) (PDLA), and poly (D,L-lactide-co-glycolide) (P(DLA-co-G) are commonly used to form the core which helps to prolong the half-life of the loaded drugs for more than 10 h [196,222]. Dendrimers are small particles with sizes less than 12 nm, composed of repeating monomeric or oligomeric units with an internal cavity surrounded by reactive terminal groups [223]. Dendrimers are used to encapsulate different drug agents, such as siRNA, and are highly efficient for BBB crossing, but are toxic to normal tissue because of their interactions with the cell membrane and their less controllable release behavior [224].

The use of nanocarriers has been well established over the past decade both in pharmaceutical research and clinical settings to enhance the in vivo treatment efficiency [225]. For GBM, such treatments are in development as well.

- Lipid-Based Nanocarriers for Glioblastoma Treatment

Lipid based NPs have been extensively used to target GBM. They can efficiently encapsulate multiple drugs that act synergistically to kill GBM cells or drugs with poor physicochemical properties (e.g., poor water-soluble drugs). For example, in a study conducted by Zhang et al., glucose-functionalized liposomes (gLTP) that co-load TMZ and pro-apoptotic peptide (PAP) can cross the BBB through the glucose–GLUT1 pathway to deliver these drugs to the tumor site. PAP affects the mitochondria reducing ATP generation, while enhancing the sensitivity of GBM cells to TMZ [226]. In the same way, Papachristdoulou et al. delivered, via liposomes, O6-(4-bromothenyl) guanine derivates (O6BTG) targeting MGMT to enhance TMZ efficacy in vitro [227]. The magnetic resonance image-guided microbubble was also used to enhance the low-intensity pulsed focused ultrasound that permits the opening of the BBB and better deliver the liposomes [227]. The O6BTG-liposomes combined with TMZ reduced the tumor growth and increased mice survival [227].

In another study, Jhaveri et al. encapsulated RES, a natural polyphenol with poor physicochemical properties, at a drug loading efficiency of around 70% in PEGylated liposomes. These liposomes were modified with Tf moieties to increase their interaction with GBM cells (Tf-RES-L) [228]. They found that liposomes prolong the drug-release in vitro, and delivered RES induces GBM cell cycle arrest at the S-phase and activates their apoptosis through caspases 3/7 [228]. Tf-RES-L also permits an inhibition of GBM tumor growth in vivo, improving survival in mice [228].

Among many types of lipid-based nanoparticles, NLCs and SLNs gained an extreme focus for developing GBM treatments, especially in the delivery of GBM-targeted ACA [196,229,230]. Song et al. prepared dual-ligand-commodified NLCs using both lactoferrin, a member of the Tf family, and RGD peptides recognized by integrins overexpressed in GBM cells. They demonstrated that the use of these dual-ligand-comodified NLCs (139 nm) for TMZ and vincristine delivery induce a higher cytotoxic effect in vitro on human GBM cells (U87MG cells) compared to single-drug-loaded NLCs or free drugs. The same results were gained in vivo on the inhibition of tumor growth in U87 MG cell-bearing nude mice [231]. Furthermore, the size of the tumor treated by dual lactoferrin/RGD-NLCs was reduced compared to the tumor treated by single-ligand NLCs [231]. The use of dual ligands improves the GBM cell targeting in the brain. However, the lipid composition of NLCs can also greatly influence the ability of the liposome to cross the BBB and target GBM cells. Zwain et al. prepared NLCs using four liquid lipids alone or in combination (propylene glycol monolaurate, propylene glycol monocaprylate, caprylocaproylmacrogol-8-glycerides, and/or polyox-yl-15-hydroxystearate) to encapsulate the poorly water-soluble docetaxel (DTX), also known as Taxotere^®^. They observed that NLCs composed of the four lipids had not only the highest drug loading (almost 89%), but they also crossed the BBB in the in vitro model without the loss of the barrier integrity [232]. These NLCs, with an average particle size of 123.3 nm, were internalized more efficiently by U87MG cells compared with non-cancerous cells. They were also more efficient to reduce the size of U87MG spheroids than the free drug, by inhibiting both the cell cycle via the G2/M phase and mitochondria activity [232]. Another strategy to target GBM cells is the use of antibodies against specific growth factor receptors such as the epidermal growth factor receptor (EGFR). For example, carmustine-loaded cationic SLNs grafted with an anti-EGFR permit and affective delivery of the drug resulting in an antiproliferative efficacy against the tumor growth [233].

SLNs and NLCs are effective as drug carriers for GBM. However, despite all the promising results in the literature, none of these carriers have been successfully developed by a pharmaceutical company. Therefore, more efforts should be focused on the development of reproducible nanocarriers.

- Polymeric-Based Nanocarriers for Glioblastoma Treatment

Due to their biocompatibility, low toxicity, and biodegradability, polymers offer many advantages [234]. Han et al., in a recent study, used paclitaxel (Taxol^®^)-loaded into dextran NPs coated with RVG29 peptide for targeted chemotherapy in glioma [235]. RVG29 is a peptide with a high affinity to the nicotinic acetylcholine receptor (nAchR) on neuronal cells and is highly implicated in drug resistance [235]. The use of NPs either in vitro or in vivo exhibited a higher cell growth inhibition rate against C6 cells compared with the non-grafted NPs [235]. To our knowledge, these polymers are not used much in the context of GBM treatment compared to other cancers such as breast cancer [236], cervical cancer [237], and lung cancer [238].

On the other side, PLGA, PLA, and PGA-based nanocarriers are the most extremely used polymers in brain delivery (Table 3) [239]. In the context of GBM, these nanocarriers are mainly used for the encapsulation of chemotherapeutic molecules to control their release. For example, DOX-loaded PLGA-PEG NPs with a size around 200 nm were prepared for the in vitro study of the drug kinetic release, no burst release was observed, and a sustained release was maintained for up to 96 h [240]. Ramalho et al. used PLGA-NPs functionalized with OX26, a monoclonal antibody for a transferrin receptor, and loaded with TMZ for targeting U215 and U87 cell lines. The NPs showed an encapsulation efficiency of 48% and a size of 194 nm, both free and encapsulated TMZ induced a decrease of cell growth in the studied lines, but the use of NPs exhibits a longer and stronger action on the cells [241]. Caban-Toktas et al. studied paclitaxel co-loaded in PLGA NPs with R-Flurbiprofen, a nonsteroidal anti-inflammatory drug known for its strong anticancer activity [242]. In the same study, chitosan-modified PLGA NPs were also co-loaded with paclitaxel and R-Flurbiprofen for an efficient delivery to the tumor site [242]. Sixty percent of the paclitaxel was released from the NPs for five days until reaching the pseudo-plateau. On the other hand, R-flurbiprofen was released quickly in the firsts 6 h. Additionally, the NPs showed efficient cytotoxic activity and were well integrated by the tumor cells, resulting in anti-tumoral activity against glioma [242]. The in vivo studies confirmed that the use of paclitaxel-loaded NPs with R-flurbiprofen-loaded NPs induces a significantly higher reduction of the tumor compared to when the drug-NPs are used individually [242].

Due to the protection they provide, NPs can also be used to encapsulate and deliver peptides and small proteins, such as CXCL12, in the aim of controlling GBM cell migration as a therapeutic approach. Our team strongly believe that the control of GBM cells migration via the CXCL12–CXCR4 axis can be a promising approach to rule the spread of those cells and facilitate their elimination. For this aim, we have developed composite alginate–chitosan NPs with an average size of 250 nm for CXCL12 encapsulation which, upon its release, increases GBM cell migration [243]. Three initial mass loadings were tested (0.372 µg/mg NPs, 0.744 µg/mg NPs, and 1.490 µg/mg NPs). Our results showed that the alginate–chitosan NPs entrapped CXCL12 with a percentage of ~98% without loss of the molecule [243]. For all the conditions tested, a burst released in the first 2 h was observed, followed by a sustained release that reach a pseudo-plateau after 72 h without a complete release of the chemokine [243]. The releasing profile observed was coherent with a diffusion-based system which led us to evaluate the driving mass transport phenomenon [243]. Using the experimental data, we performed mathematical modeling using Fick’s second law of diffusion for a spherical geometry, which considered the size distribution of NPs (class method) and boundary conditions that allowed us to model the interactions between the CXCL12 molecules and the NPs. The cumulative mass release vs. time and position equation was solved using a finite difference approach and mechanistic parameters (effective diffusion coefficient, D_eff_; overall mass transfer coefficient at the surface, k) which were estimated using an evolutionary algorithm, leading to coefficients of a determination > 0.97 [243]. Small values of D_eff_ (~2 × 10^−19^ m^2^/s) were obtained, which can be associated with the presence of electrostatic interactions between the positive charge of CXCL12 and the negative one of the alginates composing the NPs [243]. However, since our experiments were conducted in static conditions, it is impossible to deny that in vivo, other types of mass transport phenomena may occur, such as convective interstitial brain fluid flow, that may be a reason for an increased releasing rate. Furthermore, our migration assays proved that CXCL12 significantly controlled the invasion of the F98 cell line, which highlights the importance of using CXCL12 in delivery systems for GBM targeting [243].

In the same way, Mansor et al. used PLGA and the PLGA-PEG co-polymer for CXCL12 encapsulation [213]. They used a different proportion of PLGA, an encapsulation efficiency of 67% was observed for 0% PLGA-COOH, and for the two other proportions (17% and 67% PLGA-COOH), the encapsulation was surprisingly above 100% [213]. The highest percentage of CXCL12 released was 43%, achieved with a 33% proportion of PLGA-COOH in a physiologically relevant solution (pH 7.4). This percentage increased when the medium was changed to an acidic medium buffer (pH 4). As another example, Alghamri et al. developed synthetic protein NPs coated with iRGD to target the CXCL12–CXCR4 axis in GBM. The NPs blocked CXCR4 via its inhibitor in both in vitro and in vivo models. The treatment inhibits GBM proliferation and the induction of immunogenic tumor cell death. Further, the use of radiotherapy with the treatment inhibited GBM progression, leading to a 60% increased survival rate compared to the controls [244].

**Table 3:** Some examples of polymeric-based NPs.

Polymer Type	Drug/Molecule Loaded	Particle Size (nm)	Ref
PLGA	DOX	~120	[245]
PLGA	TMZ	~194	[241]
PLGA-PEG	DOX	~50	[240]
PLGA-PEG-chitosan	Paclitaxel and R-flurbiprofen	150–190	[242]
PLGA	DTX and indocyanine green	~220	[246]
PLGA/PEG-PLGA	CXCL12	200–250	[213]
Chitosan-Alginate	CXCL12	~250	[243]
Chitosan-modified PLGA NPs	R-Flurbiprofen and Paclitaxel	150–190	[100]
Dextran	Paclitaxel	~60	[235]
Silk fibroin	Indocyanine green	~209	[247]
Synthetic protein	AMD3100	37–98	[244]

NPs provide a series of advantages for delivery applications to enhance the therapeutic efficiency of the drugs, but the major challenge remaining is the transport to the tumor site without degradation [248]. Despite all the advantages of systemic drug delivery across the BBB, systemic delivery needs to address different challenges for further improvement. The long distance between the delivery route and the target site and the drug digestion remain the biggest limitations of systemic delivery.

#### 6.2.2. Local Delivery

Local delivery consists of delivering chemotherapeutic agents in the surgical cavity after the tumor resection for GBM therapy improvement [41]. A series of factors make local delivery advantageous in GBM treatment [249]. Metastasis occurs within approximately ~2 cm of the tumor’s original site, which means it is close to where the drugs were loaded locally; the administration of the drugs will no longer face the BBB limitations [249]. Different strategies for local delivery to improve the GBM survival rate have been developed, including intranasal drugs delivery, convection enhanced delivery, and direct injection of the drugs including rigid implants and hydrogels [250].

Intranasal Drugs Delivery:

The intranasal route can bypass the BBB and achieve reaching the brain for the delivery of drugs. It is important to understand the anatomy of the nasal cavity and the mechanisms of compound transport through the intranasal route. Bruinsmann et al., described this aspect in detail [251]. In this paragraph, we focus on discussing the different GBM treatments via the intranasal approach to deliver drugs to the tumor.

Blacher et al. used “anthranoid 4,5-dihydroxyanthraquinone-2-carboxylic acid”, also known as rhein, to inhibit CD38 by intranasal injection in mice. CD38 deficiency is known to regulate microglial activation and attenuates glioma progression [252,253,254]. The tumor in the mice treated decreased, concluding that the intranasal drugs’ administration is effective and rhein can be a therapeutic target in GBM [252]. Li et al. used intranasal drug delivery to administer TMZ in a rat model bearing orthotopic C6 glioma xenografts showing a significantly reduction in the tumor growth compared with intravenous injection or gavage [255]. The results suggest that the intranasal route should be further considered as an option for TMZ delivery into the brain [255]. However, different therapeutic agents are under investigation for GBM treatment, but the use of a delivery system has been proved to be more beneficial in term of maintaining the drug release.

NPs are also good vehicles to control the drug release and overcome some limitations of the intranasal drug delivery, such as the poor capacity of crossing the nasal mucosa and enzymatic degradation [251]. Polymeric NPs (PLGA-based [251,256], PCL-based [257,258]) and lipid-based NPs are the most commonly used for nose-to-brain delivery [196,259]. For instance, PLGA and oligomeric chitosan composite NPs were designed to co-deliver alpha-cyano-4-hydroxycinnamic acid (CHC) and the monoclonal antibody cetuximab (CTX) into the brain by nasal administration to ensure the therapeutic efficacy for GBM treatment [256]. CHC and CTX are known to have a therapeutic effect against angiogenesis, cancer cell invasion, and metastasis [256]. In vitro assays using a chicken chorioallantoic membrane assay showed no reduction of cell viability for U251 and SW1088 glioma cell lines, but the designed NPs showed a stability that reached three months and a high encapsulation of the drugs was reached [256]. More recently, they designed a new platform using PLGA and chitosan composite NPs to carry CHC. CHC-NPs were covalently coated with CTX. An ex vivo study using a porcine mucosa demonstrated the capacity of the NPs to promote CHC and CTX permeation, whereas the chicken chorioallantoic membrane assay demonstrated its capacity to reduce the tumor size [260]. Sousa et al. also used PLGA-based NPs to administer the monoclonal antibody bevacizumab, an anti-VEGF used as an anti-cancer drug, intranasally in mice [261]. The use of bevacizumab-loaded NPs when administered intranasally into CD-1 mice showed higher brain bioavailability compared to the free drug. Furthermore, used in a GBM nude mouse model, the NPs-based delivery system also induced a reduction in the tumor growth after 14 days, with a high anti-angiogenic effect of bevacizumab compared to free drug administration [261]. PCL-based NPs have also been used to route drugs addressing the brain tissue intranasally. De Oliveira et al. used PCL based NPs loaded with melatonin to target the U87 MG GBM cell line [258]. The NPs revealed to be non-cytotoxic on healthy cells (MRC-5) and increased the water solubility of the drug in addition to promoting strong activity against U87 MG cells. In vivo assays in rats through intranasal injection increased the drug uptake in the brain compared to when administrated directly without nanocarriers [258]. Conversely, Alex et al. develop PCL-based NPs to encapsulate the anticancer drug carboplatin to target GBM via the nasal route [257]. The optimized formulation was a 311.6  nm particle size, and they observed a burst release of the drug followed by a slow continued release. However, ex vivo permeation studies through sheep nasal mucosa showed a lower drug permeation, which was attributed to the nasal mucosa complexity. While improving the delivery and accumulation of drugs to the brain, those results highlight the complexity of the nose-to-brain route. Despite all the advantages given by intranasal delivery, the low volumes of the drugs delivered remain the main problem that limits its use.

Convection Enhanced Delivery:

Convection enhanced delivery (CED) is a local therapeutic method that aims to enhance intracerebral drugs diffusion to the CNS by bypassing the BBB, allowing the introduction of high doses of therapeutic agents with different ranges of molecular weight through the interstitial spaces [262]. CED is based on the principles of “bulk flow” which refer to the extracellular flow of fluid delivered via a pressure gradient rather than the normal passive diffusion transport [262]. CED fundamental procedures consist of the stereotactic placement of a microcatheter directly into the tumor and generating an external pressure using a motor-driven pump to induce fluid convection in the brain [263]. CED permits a deeper penetration and distribution of the drugs, eliminating the problem of depletion frequently seen using the direct injection [264]. In addition, CED is used even with agents with a high molecular weight, including proteins, nucleic acids, and antibodies [265,266].

Several studies have proved the safety and feasibility of CED [106,263,267]. Additionally, drug encapsulation into nano-sized carriers proved to be more beneficial to increase the efficiency of delivery. For instance, Séhédic et al. developed lipid nanocapsules (LNCs) to incorporate radionuclides and implant them in the brain using stereotactic injections for locoregional therapy [268]. Using the CED, they demonstrated that lipophilic thiobenzoate complexes of rhenium-188 loaded in LNCs (LNC188Re) with a function-blocking antibody (12G5) directed at the CXCR4 on its surface enhance the median survival and show major clinical improvement in Scid mice [268]. The retention of rhenium in the brain and the outcomes achieved (distribution, efficacy, gradient) were principally ensured by LNCs, which accentuate the interest of using nanocarriers. Zhang et al. used cisplatin-loaded NPs of 70 nm in diameter functionalized with PEG for administration by CED to control the release of cisplatin and kill the tumor cells that they reach without causing toxicity [269]. Their small size and dense PEG corona prevented them from being trapped as they moved within the brain tissue while controlling the delivery of the drug, making them efficient brain-penetrating drug delivery vehicles. Their results also showed a significant increase in the survival rate of a GBM rat brain tumor model, thus highlighting the advantages of using NPs. CED, combined with the delivery of the favorable physicochemical properties ensured by NPs, has demonstrated a great potential to improve clinical outcomes. For instance, Stephen et al. used magnetic NPs coated with a chitosan-PEG copolymer to deliver MGMT inhibitor O6-benzylguanine via CED for GBM targeting as a treatment for GBM patients showing resistance to TMZ [270]. They showed that the distribution of the NPs in the mice’s brains was excellent, whereas the activity of MGMT decreased significantly, which, in the presence of TMZ, increased the median survival rate [270]. In another study, Chen et al. proved that the nanoliposomal formulation of irinotecan with CED technology enhanced the survival time of the treated mice when combined with radiation, as compared with the systemic injection of irinotecan plus radiotherapy [271]. CED for GBM treatment has been also reported in clinical trials. Cruickshank et al. injected irinotecan drug-loaded beads suspended in an alginate solution into patients after surgical resection. Studies are still under investigation, however, only one patient has died, due to causes which were not associated with the treatment, thus suggesting that the use of irinotecan drug-loaded beads may be a promising, stable, and safe platform to assess the local delivery of new agents [272]. All these studies focus on combining CED with nanocarriers for a better control of the drug release in the aim of increasing survival. Despite the promising results achieved, different physical and technical limitations and challenges are still to be overcome. The main obstacle occurring is the backflow, sometimes referred as reflux, that takes place when the perfusate is not well penetrated in the tissues [262]. Hopefully, backflow resistant catheters have been developed, which may solve this issue.

Direct Drug Injection:

The direct drug injection for the delivery of ACA within the tumor resection cavity emerged to resolve the bypassing BBB limitations and increase the drugs’ concertation in the tumor site. This method has lot of advantages, including side effects reduction, safe administration of different molecules, and the depletion of toxicity actions [273].

- Rigid Implants:

The direct drug injection opens the doors for local implant-based GBM treatments. The only local delivery system approved by the FDA and currently used for GBM treatment is the polifeprosan 20 with the carmustine-loaded wafer Gliadel^®^ [274]. Gliadel^®^ wafers are 14.5 mm in diameter and 1 mm in thickness, the wafer is made from a biodegradable hydrophobic co-polymer 1,3-bis-(p-carboxyphenoxy)propane (pCPP) and sebacic acid [274]. When in contact with aqueous fluids, the wafers start releasing the carmustine into the surrounding tissue [275]. After approving Gliadel^®^ wafers, several chemotherapeutic agents have been tested in preclinical models such as paclitaxel [276], acriflavine [277], and DOX [278]. Due to the rigidity of the device compared with the soft nature of the brain tissue, different adverse reactions occur such as necrosis, infection, and convulsions [277]. The main problem remaining using these wafers is the drug release profile. For this, many studies have explored the possibility of reducing the burst release profile and sustaining the release for a long period [250,279,280]. For example, Shapira-Furman et al. used Gliadel wafers co-loaded with 50% *w*/*w* of TMZ and BCNU in PLGA for sustaining the release of the drugs for four weeks [281]. The drugs were first coated with the polymer to form core–shell particles, in which the coating shell served as a membrane for the drug particles [281]. The median survival was 15 days in the group treated with BCNU wafers alone, whereas the group with TMZ wafers alone had a median survival of 19 days [281]. The group treated with combined BCNU and TMZ wafers had a median survival of 28 days, suggesting that the combination of drugs can achieve a big improvement for local drug delivery [281]. However, rapid drug release, cell migration, drug resistance, and side effects are different problems that prevent Gliadel wafers from being an effective option to treat GBM.

- New Innovative Drug-Delivery Approaches in Glioblastoma Treatments:

Hydrogels:

Hydrogels can be defined as 3D polymeric hydrophilic networks within an aqueous medium [282]. Due to their ability to encapsulate different agents and control their release, hydrogels are used for different biomedical applications and medicine such as artificial skin, membranes for biosensors, 3D platforms, and drug delivery devices [283,284,285]. Further, the use of hydrogels can be more beneficial than the Gliadel wafers because of their ability to mimic the mechanical properties and the softness of the brain tissue. For instance, Wang et al. have shown, using PEG-based hydrogels bearing GRGDS adhesion peptides and U87 human GBM cells, that matrix stiffness induces differential GBM cell proliferation, morphology, and migration [286]. Increasing the matrix stiffness (associated with tumor-like mechanical properties) led to delayed U87 cell proliferation, but the authors observed that cells formed denser spheroids with extended cell protrusions.

Various studies explored the use of hydrogels for GBM treatments in this context [286,287,288,289]. Bastiancich et al. reviewed the different types of hydrogels as drug delivery systems for GBM local treatment recently used in preclinical and clinical studies, suggesting that loaded hydrogels with one or many chemotherapeutic agents are advantageous for GBM treatment [290]. Hydrogels fill the gap between the tumor resection and the administration of chemotherapy and radiotherapy, allowing a sustained release of the drugs, which may lead to better results than a conventional CED approach [290]. Akbar et al. developed a biodegradable hydrogel from PLGA:plasticizers with a ratio of 40:60 for TMZ delivery in C6 glioma rats [291]. A significant reduction of the tumor was observed, and no mortality was associated with the gel matrix treatment, concluding that the gels can be safe and effective when used in vivo [291]. In another recent study, hydrogel loaded with the quisinostat drug and radiopaque gold NPs (AuNP) has been explored in GBM. Radiopaque NPs were used as the contrast agent that would release the drugs when irradiated. The release of quisinostat in vitro was high, which inhibited the tumor growth in the in vivo mice model bearing xenografted human GBM tumors [292]. The platform developed can also be used simultaneously for radiation therapy. OncoGel™ is a PLGA-PEG-based thermo-sensitive hydrogel used for paclitaxel delivery in GBM treatment, which has been shown to prolong the survival in a rodent glioma model [293]. OncoGel™ provides a sustained release of paclitaxel for 50 days, maintaining a high local concentration and biodegrades after four to six weeks [294]. In 2007, the first clinical trial (NCT00479765) using OncoGel™ for recurrent glioma in order to evaluate the safety and tolerability of the system in the patients started, but could not be ended for sponsor businesses [290]. Déry et al. used a biodegradable hydrogel (GlioGel) loaded with three chemoattractants (CXCL10, CCL2, and CCL11) to attract murine F98 and U87 GBM cells toward a therapeutic trap using an agarose drop assay [295]. The zones with high concentrations of CXCL10 display the highest number of the cells attracted compared to the control due to the chemoattractant gradient. CCL2 showed a very similar response to CXCL10 for the F98 cells, but the U87MG cells were less responsive, and both cells did not show any significant effect on chemotaxis to CCL11 [295]. The team performed in vivo assays using an orthotopic syngeneic F98-Fischer rat model. Three days after the implantation of the F98 tumor cells, the GlioGels containing the chemoattractant were inoculated. Many peritumoral clusters were observed when CXCL10, CCL2, and CCL11 were implanted in the controlateral hemisphere compared to those implanted in ipsilateral hemisphere [295]. These results support the hypothesis that the use of the GlioGel with chemokines can modify the migration behavior of the GBM cells.

Nonetheless, for a sustained controlled release of chemotherapeutic drugs, nanocarriers can be confined in a hydrogel. Several reviews have highlighted that a better controlled release can be obtained when NPs are loaded in hydrogels [296,297]. For instance, Brachi et al. have shown that a multi-component system composed of polymeric NPs BODIPY-loaded and embedded within a thermosensitive hydrogel, revealed to be more efficient in terms of drug retention within the tumor in an orthotopic GBM mice model compared to NPs alone [298]. Zhao et al. used PLGA NPs for paclitaxel encapsulation, then loaded them into photopolymerizable hydrogels that had been implanted in the resection cavity [299]. They found that the system enhanced long-term survival (<150 days) in the U87 cells in vivo mice model compared with the mice where the hydrogels were implanted empty (they tolerated the hydrogels and had a long healthy life for up to four months) [299]. Furthermore, for biocompatibility, biodegradability, a better control of the gelation time, and a sustained release of hydrophobic and hydrophilic drugs, some prepolymer hydrogels have shown remarkable results, but still need to be investigated in GBM [300,301,302]. However, because of the many advantages of combining NPs to hydrogels, this approach can also be transposed to the development of a chemoattractant releasing device as part of cancer cell traps.

**Figure 6:**
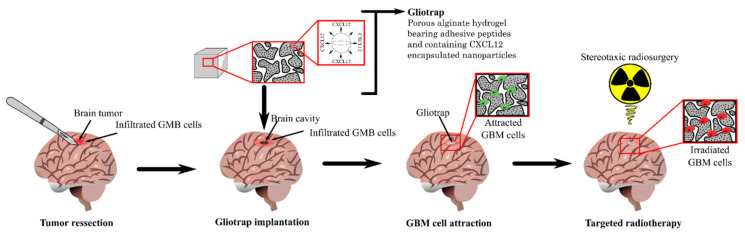
The strategy proposed by our team for GBM treatment.

Cancer Cells Trap:

Najberg et al. proposed, in 2019, trapping cancer cells within one confined area to facilitate their removal [297]. In the paper, different strategies for cell trapping, such as the use of chemotaxis to attract cancer cells expressing CXCR4, were proposed [297]. Giarra et al. used this technique for preparing a fake metastatic niche made of CXCL12-loaded thermoresponsive hydrogels based on methylcellulose or polaxamers with or without HA [303]. Only gels based on methylcellulose embedded with CXCL12 allowed GBM CXCR4-expressing cells to migrate in the direction of the hydrogel and finally be captured within [303]. They also demonstrated that the cancerous cell migration depended on the mechanical properties of the hydrogel; softer hydrogel made of methylcellulose promotes better cell migration as opposed to stiffer poloxamers hydrogels [303]. Similarly, Molina-Peña et al. loaded CXCL12 in PLGA and PEG-PLGA NPs to integrate the system into a chitosan solution with fiber-forming additive polyethylene oxide for producing a nanofibrous scaffold by electrospinning [304]. The use of the nanofibrous scaffolds with the NPs allowed a sustained release for up to 35 days instead of 5 days for the NPs alone, and an attenuation of the burst release [304]. They have also assessed this system in vivo with blank-NP-loaded nanofibrous scaffold and found no difference between the rats with the scaffolds implanted and the controls, suggesting an excellent biocompatibility of the scaffold during the first week of the treatment [304]. In the same context, our research group has recently developed an alginate-based macroporous hydrogel as a potential device for GBM cell trapping [29]. Since the main cause of GBM recurrence is the infiltrated GBM cells that migrate from the tumor, as discussed earlier, we propose to inverse the direction of GBM cell migration towards a well confined area in which they will be trapped and can be eliminated with localized radiotherapy using a chemoattractant gradient of CXCL12 (Figure 6).

The device proposed takes the name of gliotrap (GBM-trapping), and it combines an alginate macroporous hydrogel functionalized with RGD peptides (for cells catching) and alginate–chitosan composite NPs encapsulating CXCL12 (for cells attraction). The functionalization of the hydrogel with RGD peptides aims to promote the GBM cell adhesion inside the matrices via the interactions between the peptides and the αvβ3 and αvβ5 integrins widely expressed by those cells, as discussed above [29]. Hydrogels will be loaded with the NPs’ delivery system and implanted into the surgical cavity of the tumor after its resection to ensure a CXCL12 gradient maintained for a very long period. The NPs are designed to promote a controlled release of CXCL12 and so create a cancer cell attracting gradient. This will help to maintain the CXCL12 gradient under a fast release that could happen because of the fluid flow in the brain.

So far, we have demonstrated using Transwell^TM^ chemotaxis assays, that the NPs loaded with CXCL12 alone are capable of increasing the invasion of F98 cells [243]. Further, we previously developed an RGD-functionalized macroporous matrix in which the GB cells can accumulate and be retained [29]. Using an F98 GB cell line, it was demonstrated that the macroporous matrix can possibly accumulate and retain the F98 cells. These cells could then be effectively eliminated using local stereotaxic radiotherapy [29]. The main challenge remaining is to combine the two systems by loading the NPs-CXCL12 into the macroporous hydrogel. The release kinetics of CXCL12-AF647 encapsulated in alginate–chitosan NPs from the macroporous matrix will be investigated in the next step. Additionally, we will investigate the effect of a simulated brain fluid flow on CXCL12 release from the porous matrices using a custom-made 3D in vitro model that considers the fluid flow impact. The conception of a 3D in vitro model that mimics the brain fluid dynamics will provide a better understanding of the impact of the interstitial fluid flow on the release profile of CXCL12 for further optimization.

## 7. Conclusions

The present review discussed different novel strategies for GBM treatment that have emerged during the last decades. The standard treatments for the disease lack specificity and are very limited, mostly due to their incapacity to target cancerous cells that migrate out of the tumor to the surrounding parenchyma. We have also highlighted, as interesting therapeutic targets, several phenomena that are known to have a strong impact on GBM cell migration, such as the CXCL12–CXCR4 axis, the ECM composition, as well as the interstitial fluid flow. Additionally, we have discussed different drug delivery systems currently used in the development of GB treatments. Systemic administration has shown to be very beneficial. The use of NPs particularly opened a new horizon as they can promote drug accumulation at the site of the tumor and have shown better results when combined with other treatments such as immunotherapies, or with another drug delivery system, such as cells. Although there is still a lot of research to do in this field, the type of drug used, its administration, and the complexity of the body’s reaction to the treatment require the development of new pre-clinical models that mimic the complexity of the human brain. Local administration of drug delivery systems, such as intranasal drug delivery, CED, and the implantation of polymer-based biomaterials (rigid implants and hydrogels), have proved to be even more effective. More precisely, hydrogels also proved to be advantageous compared to the rigid implants because of their unique characteristics, including biocompatibility, biodegradability, and their response to stimuli. Hydrogels can be implanted into the surgical cavity after the tumor resection to ensure a high local concentration of the therapeutic drug and a sustained release in the tumor site, especially when combined with NPs. New strategies in drug delivery devices addressing GBM are also focusing on the development of GBM cancer cell traps, which comprises of a hydrogel with embedded NPs used to trigger cell chemotaxis, while the hydrogel itself acts as a cell capturing device. Finally, we highlighted with our ongoing research that, as promising as these new technologies are, there are still many challenges remaining.

## Figures and Tables

**Table 1:** Some activated axes in GBM and their actions.

Axis	Actions in GBM			
	Migration and Invasiveness	Proliferation	Growth, Survival, and Apoptosis	Ref
CXCL12–CXCR4	Chemotaxis	Activation of Ras, Raf kinase	Ca^2 +^ mobilization via inhibition of cAMP (survival)	[91]
		ERK1/2 phosphorylation		
CXCL12–CXCR7	Activation of β-arrestin by heterodimerization with CXCR4		Activation of ERK1/2 via GRK	[92,93]
	Activation of ERK1/2			
CXCL8–CXCR1-2	Overexpression of MMP-9 and MMP-2	High density of macrophage promotes a high degree of microvascular proliferation	Activation of IL-6	[94]
			Activation of JAK pathway	
	EMT transition		Increase of anti-apoptotic protein secretion	
CXCL16–CXCR6	Overexpression of anti-inflammatory genes and modulating microglia polarization		Establishing a pro-tumoral microenvironment in the brain	[95]
	Increase of MMP-9 and MMP-2 expression			
CCL5–CCR5	Activation of Akt kinase		Stimulation of AKT pathway	[94,96]
CX3CL1–CX3CR1	Modulation of the activation of TGF-beta1	Not clear	CX3CR1 polymorphism through isoleucine V249I (survival)	[97,98]

Ca^2+^: Calcium; CXCR7: receptor C-X-C type 7; GRK: G protein-coupled receptor kinases; CXCL8: C-X-C motif chemokine ligand 8; CXCR1-2: C-X-C chemokine receptor 1 and C-X-C chemokine receptor 2; CXCL16: C-X-C motif chemokine ligand 16; CXCR6: C-X-C motif chemokine receptor 6; CX3CL1: chemokine [C-X3-C motif] ligand 1; CX3CR1: C-X3-C motif chemokine receptor 1; CCL5: C-C motif chemokine ligand 5; CCR5: C-C chemokine receptor type 5; IL-6: Interleukin 6; TGF-beta1: transforming growth factor beta 1; EMT: epithelial–mesenchymal transition.

**Table 2:** Advantages and disadvantages of different models used in the study of GBM.

GBM Study Models			Advantages	Disadvantages	Applications	Ref
2D	In vitro	Scratch assays	• Easily implemented • Low cost • Real-time cell tracking	• Low physiological relevance • Migration and invasion are not distinguished	Migration Matrix remodeling Drug screening	[137]
		Transwell assays	• Technically easy • Low cost • A matrix can be added to study ECM	• Lacks tumor complexity • Lack of 3D environment without matrix • Difficult to distinguish between migration and invasion		
3D		3D bioscaffolds (Spheroids)	• Simple • Control of spheroids size • Control of growth parameters • High throughput • Allow ECM interactions study • Allow pathways signaling study	• Long-term culture difficulties • Lack tumors complexity • Lack vascularization	Migration/Invasion Cell biology (proliferation, apoptosis, etc.) Drug Screening	[138]
		Microfluidic co-culture	• Real-time cell tracking • Allow ECM interactions study • Allow pathways signaling study • Cell–Cell interactions study	• High cost • Lack of native microenvironment • Lack of tumor complexity	Migration/Invasion Cell adhesion	[139]
	Ex vivo	Organotypic brain slices cultures	• Native ECM composition • Real-time cells tracking • Maintain tumor heterogeneity • Allow ECM interactions study • Allow cell signaling study	• Ethical issues associated with animal studies • Lacks blood • Flow • Lack of tumor complexity	Migration/Invasion Tumor growth Study of drugs delivery	[140]
	In vivo	Orthotopic xenograft	• Native microenvironment • Studies under the effect of the flow • Cell–Cell interactions study • Allow pathway’s signaling study	• Ethical issues associated with animal studies • High cost • Less experimental control • Time consuming	Migration/Invasion Signaling Study of drugs delivery Tumor growth	[141]

## Data Availability

Not applicable.
